# Alternatives for fetal bovine serum (FBS) and sustainability of milk derivatives in cell culture

**DOI:** 10.1186/s40659-026-00691-0

**Published:** 2026-03-28

**Authors:** Olga Maria Pittino, Maria Sofia Semprini, Lac2Lab, Francesca Ruzzi, Stefania Angelicola, Chiara Cappello, Laura Scalambra, Mariateresa Frascino, Patrick L. S. Michel, Patrizia Nanni, Pier-Luigi Lollini, Arianna Palladini

**Affiliations:** 1 Laboratory of Immunology and Biology of Metastasis, Department of Medical and Surgical Sciences (DIMEC), University of Bologna, Bologna, Italy; 2 Lac2Lab s.r.l.s., Bologna, Italy; 3 Medical Oncology, IRCCS Azienda Ospedaliero-Universitaria di Bologna, Bologna, Italy; 4 Center of Applied Biomedical Research (CRBA), University of Bologna, Bologna, Italia; 5 Department of Molecular Medicine, University of Pavia, Pavia, Italy; 6 Medical Oncology Division, IRCCS Policlinico San Matteo, Pavia, Italy; 7 IRCCS Azienda Ospedaliero-Universitaria di Bologna, Bologna, Italy

**Keywords:** FBS, Serum substitutes, Cell culture media, Sustainable alternatives, Milk-derived components

## Abstract

Fetal bovine serum (FBS) is frequently used as a media supplement in cell cultures, as it contains many nutrients, growth factors, and hormones crucial to sustaining cell growth in vitro. However, over the years, concerns about its use have been highlighted. The unknown composition, batch-to-batch variability, and increasing demand and prices have raised scientific and economic issues. In addition, the collection procedure has raised ethical concerns. These issues have prompted researchers worldwide to explore more sustainable and reproducible alternatives performing like FBS. In this review, we examine several potential FBS substitutes, derived from other animal sources or chemically defined sera. Among the considered alternatives, milk emerges as a relevant and suitable option, helping to steer the biotechnological progress towards a more ethical and sustainable approach. Food waste is a globally widespread problem, and milk is no exception. Its use as an alternative supplement for cell cultures could contribute to a reduction in food waste and in the FBS usage, limiting the drawbacks associated with it. Furthermore, milk is often referred to as the “perfect food” as it is rich in nutrients fundamental to sustaining growth, resembling FBS characteristics. Bovine milk derivatives have proven effective in reducing and, in some cases, fully replacing FBS, therefore representing an interesting and feasible option as supplements for culture media. Considering that, requalified milk could help address both social and economic needs and, through circular economy strategies, reduce costs for the biotechnological industry and enhance environmental sustainability.

## Background

A great breakthrough in the biology and medicine fields was the development of cell culture, introduced in the 20th century. Cell culture is a technique that allows the growth of various cell types in a controlled environment, outside of their natural biological background [1]. In these conditions, it is possible to study basic cell biology, including metabolism, gene expression, protein production, and cell signaling, both in a physiological and pathological context [2]. Moreover, cell cultures can be used to test drugs’ efficacy and toxicity and to develop vaccines [3]. The use of cell culture is expected to increase in the coming years, driven both by its profound impact on human health and by the need to further limit the use of laboratory animals in accordance with the 3Rs (Replacement, Reduction, and Refinement) principle devised by Russell and Burch to improve animal welfare [4]. Nonetheless, cell culture media used in vitro typically is based on FBS, an animal-derived product; therefore, its complete replacement must be recognized as a key point within the 3Rs framework [5]. The first attempt at in vitro cultivation dates to the end of the 19th century. Since then, researchers have tested and improved various solutions containing the elements necessary for cell culturing. The Locke-Lewis solution, developed in the 1910s, was the first one to include animal blood derivatives. From this point, animal blood serum has been used as a key supplement of cell culture media, even if its exact composition is still unknown [3].

The use of FBS in cell culture media is progressively raising critical issues, including ethical, cost, and standardized production concerns. Nutrient-enriched solutions as an alternative to FBS have been formulated using components derived from animal, plant, and synthetic sources. These solutions present limits and are not always able to overcome the FBS-related issues. However, they represent a step forward for a more ethical, reproducible, and sustainable cell culture practice. This review aims to present the most common alternatives to FBS implemented in the biomedical field, with particular attention to milk whey.

## FBS characteristics and problematics

### Production of fetal bovine serum

Fetal bovine serum is a common supplement employed in cell culture [6]. Specifically, it is a derivative of the beef and dairy industry obtained from bovine fetuses’ blood. According to Lee et al. [8], this process would be performed in government-approved facilities, where generally cows and bulls graze together [7, 8]. In European facilities, about 16% of dairy cows are slaughtered while pregnant, with 3% being slaughtered in the last third of gestation. The butchery of pregnant cows can occur for various reasons, including missed pregnancy diagnosis in extensive systems, as well as health, welfare, and economic benefits [9]. When a cow is found pregnant, its slaughter usually takes place in a room isolated from the rest of the slaughterhouse. The fetus is removed aseptically, and its blood is obtained via cardiac puncture without anesthesia. Specially trained personnel are required for this procedure [7]. The obtained blood is then refrigerated to allow coagulation, centrifuged to remove cells and clotting factors, and the resulting serum is triple-filtered (0.1 μm) and eventually gamma irradiated to ensure sterility [8]. About half of the serum is lost throughout this process, implying that multiple fetuses are needed for a single liter of FBS [7, 10]. To provide an idea of the scale, 150 ml of FBS are obtained from a 3-month-old bovine fetus, 350 ml from a 6-month-old fetus, and 550 ml from a 9-month-old fetus [7]. Overall, it is estimated that the annual global production of FBS ranges between 500’000 and 800’000 L [11]. It remains unclear whether the fetus is conscious during the obtaining process. If so, it could experience considerable discomfort and/or pain, raising serious ethical concerns [6].

### FBS constituents and cell proliferation

Given its origin, FBS contains a plethora of vitamins, hormones, proteins, inorganic salts, and growth and adhesive factors that allow cells to anchor and proliferate in vitro [6]. Their concentrations vary with factors such as fetal age, sex, health conditions, and maternal diet, contributing to batch-to-batch variability [12].

Bioactive molecules include various adhesive proteins such as fibronectin, laminin, and fetuin [8, 13]. Fibronectin and laminin are glycoproteins that play a crucial role in cellular adhesion, binding several cell surface receptors, primarily integrins [14]. Fetuin, on the other hand, contributes to cell adhesion through its interaction with annexins [15]. This protein accounts for approximately 32% of serum proteins, and its concentration increases during gestation, while decreasing in adults [13, 16].

Among the bioactive molecules, the most representative protein is albumin, which constitutes up to 60% of the total protein content in serum and is a fundamental carrier of small molecules, such as lipids, salts, fatty acids, and toxic metals deriving from the medium. It can also bind growth factors and hormones, contributing to the overall mitogenic activity of the hormones themselves. This protein is also essential to maintain blood oncotic pressure and pH [17].

Several growth factors were also identified in FBS, such as epidermal growth factor (EGF), insulin-like growth factor I (IGF-I), insulin-like growth factor II (IGF-II), transforming growth factor β1 (TGF-β1), transforming growth factor β2 (TGF-β2), and platelet-derived growth factor (PDGF). These factors effectively promote cell growth and proliferation. Various cytokines are present in FBS, like interleukins, although their concentrations remain undefined [8]. In the family of globulins, α1-antitrypsin and α2-macroglobulin play important roles in reducing shear stress and stopping the action of proteases, such as trypsin, commonly used to detach and subculture adherent cells [12].

### Biological limitations of FBS

On the contrary, some other elements found in the serum can interfere with cell culturing [18]. Extracellular vesicles (EVs) are lipid-bilayer-enclosed structures released by most mammalian cells. Their functions remain partly unclear, prompting increasing interest in recent years. EVs can load different macromolecules - such as DNA, RNA, lipids, and proteins – and deliver them to specific target cells, thereby mediating intercellular communication [19]. Given these biological roles, the employment of FBS raises concerns as exogenous EVs derived from FBS could contaminate those released in vitro, interfering with the studies [18].

In addition, the risk of microbiological contamination of FBS is still a possibility. Toohey-Kurth and colleagues underlined the appalling frequency of contaminated FBS in commerce. They tested 20 batches of commercial FBS from 12 different suppliers for viral contamination and found that only one batch was virus-free. The other batches were all contaminated by 1 to 11 viruses, among which the most common was bovine viral diarrhea virus (BVDV) [20].

### Market and production challenges of FBS

Beyond the intrinsic limitations of FBS itself, the geographic origin of the raw material also poses challenges. Since the 1980s, FBS production has been largely concentrated in North, Central and South America, and Oceania, with Europe contributing to a lesser extent [21]. Notably, New Zealand, Australia, and the USA together account for 45% of global FBS production [22]. Being an animal derivative, the importation of serum is generally restricted due to the potential presence of viruses capable of crossing the placental barrier between cow and fetus [23, 24]. Consequently, commercial FBS undergoes viral testing, which significantly contributes to its cost. Nonetheless, serum price is also influenced by the epidemiological status of producing countries; FBS from regions with low viral incidence, such as New Zealand, is typically more expensive. Finally, the lack of standardized international guidelines increases the risk of transmitting untested viruses, as not all countries require the same screening protocols. Moreover, FBS prices are influenced by meat market trends and the availability of pregnant cattle, which depends on the climate conditions and agricultural policies [21, 25]. These factors highlight how external dynamics and loosely regulated systems contribute to the massive price fluctuations observed in the FBS market [26].

## New perspectives in cell culture media

Currently, the scientific community is encouraging the development of the 3Rs initiatives, including the replacement of animal experiments with in vitro methods, when feasible. However, this shift implies an even greater reliance on FBS, which could be seen as an inherent contradiction [6]. Given the challenges linked to the production, cost, and usage of FBS, researchers are seeking other alternatives in cell culture [26]. Different kinds of animal-origin sera can be found on the market, depending not only on the animal from which they are derived, but also on their age. In particular, bovine serum can also be derived from newborn calves (newborn calf serum, NBCS) or adult cows [26].

Efforts to identify effective substitutes for animal serum date back at least to 1958, when Robert W. Pumper described the adaptation of a mouse cell line to a serum-free medium [27], and have increased throughout the years, becoming a common purpose among researchers from academia and biotechnology companies [28, 29]. Serum replacements can be divided based on their composition into serum-free media, xeno-free media, and chemically defined media [29]. A possible definition of serum-free media is a supplement formulated without serum, although it may still contain animal-derived elements (serum albumins, hormones), whose concentrations are not known [30]. On the other hand, a xeno-free medium does not include animal-derived elements, but it is not necessarily human serum-free [29]. Lastly, chemically defined media are characterized by a well-defined composition, where all the factors are identified and quantified. Generally, they do not contain any animal or human molecules and are serum-free [31].

It is possible to monitor the development of new supplements on the website https://fcs-free.sites.uu.nl/database/ (accessed on 22nd of November, 2025). To this day, 1180 cell lines have been cultured with serum-free media [32].

**Fig. 1:**
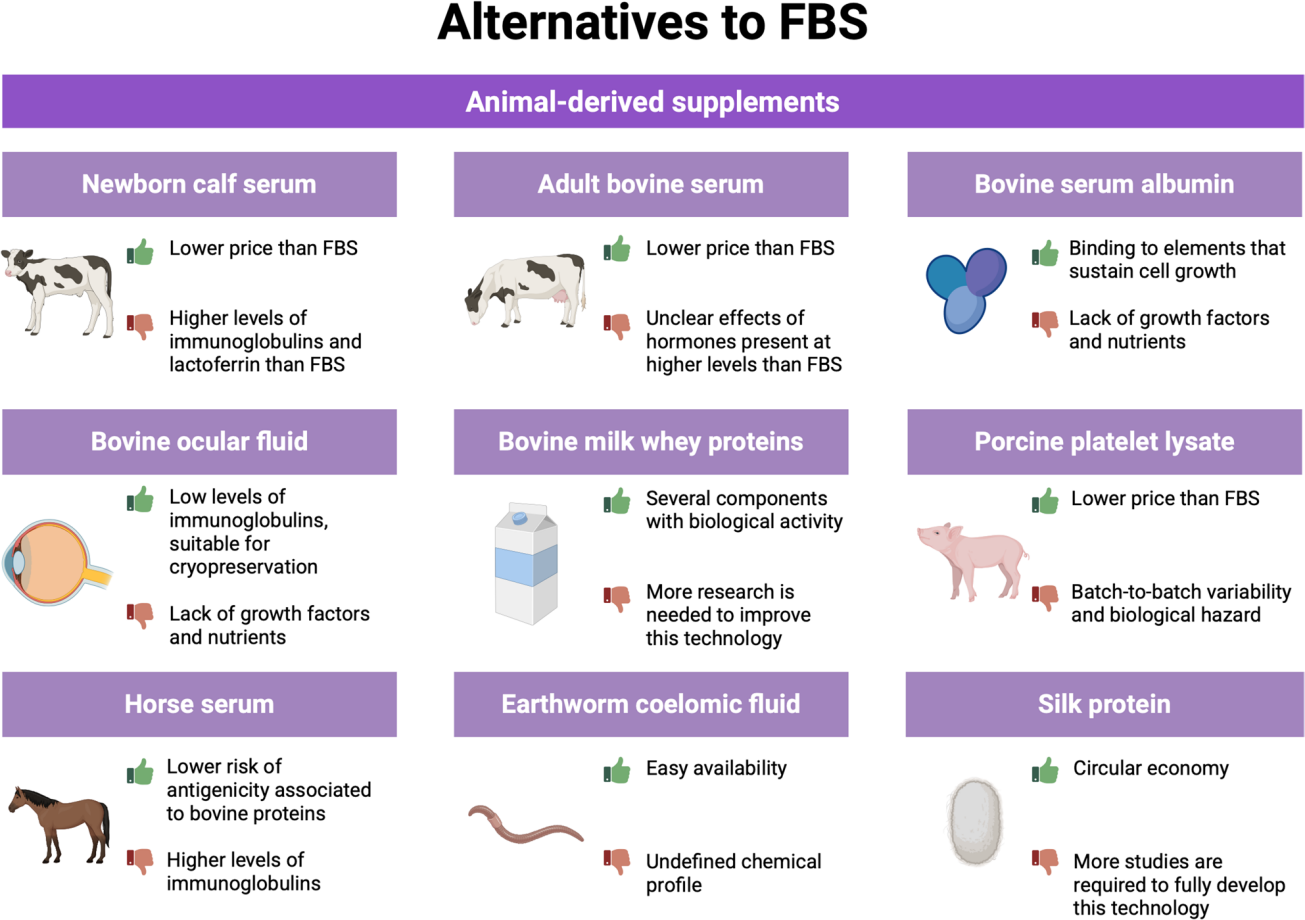
Examples of animal-derived supplements that are used nowadays as substitutes for FBS (fetal bovine serum). For each one, advantages (green thumb up) and disadvantages (red thumb down) of their use are reported (created with Biorender.com)

**Fig. 2:**
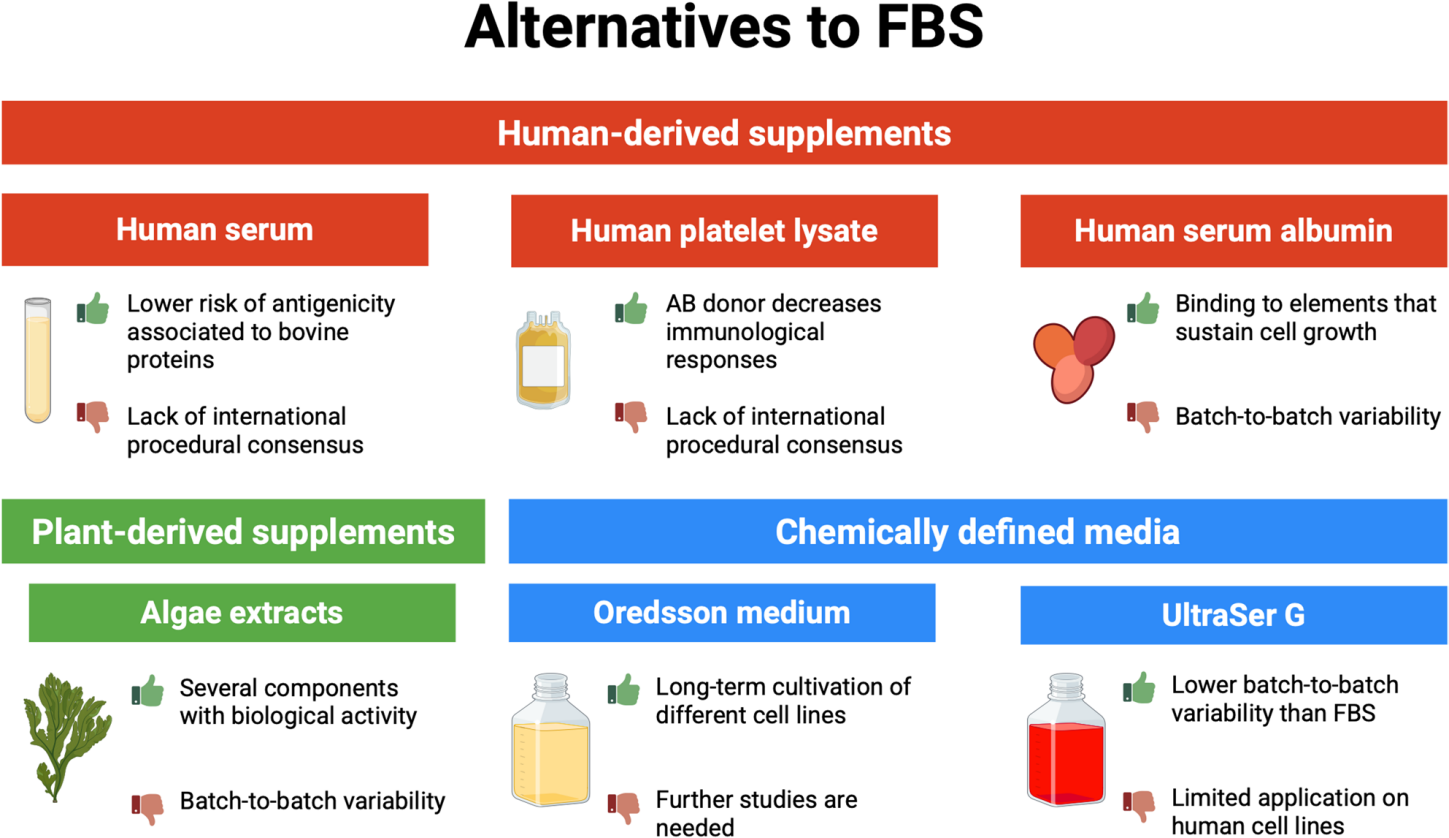
Examples of human-derived, plant-derived, and chemically defined media that are used nowadays as substitutes for FBS (fetal bovine serum). For each one, advantages (green thumb up) and disadvantages (red thumb down) of their use are reported (created with Biorender.com)

The most used alternatives to FBS will be illustrated below, considering separately animal-derived supplements (Fig. 1), human-derived, and plant-based supplements, or chemical additives (Fig. 2).

### Media obtained from animals

#### Newborn calf serum

Similarly to FBS, newborn calf serum (NBCS) is obtained from bovines after birth. They are slaughtered in controlled abattoirs, the blood is collected, and the serum is extracted after coagulation. The bovine calves are generally not older than 20 days. In this period, they are fed with colostrum, explaining why the characteristics of their blood are different from those of the fetuses [33]. FBS and NBCS differ substantially in the levels of immunoglobulins, where the most represented in NBCS is IgG1. A few weeks before delivery, the colostrum is enriched with immunoglobulins through receptor-mediated transport from the serum to the mammary gland of the cow [34]. Through this process, called passive transfer, the immunoglobulins are transferred from the dam to the calf, which absorbs IgG1, IgA, and IgM through the digestive mucosa and the Fc fragment receptor [35]. However, the levels of these components are variable, and many factors could be responsible for this fluctuation. For example, the age of the dam, the time and volume of colostrum ingestion, and the immunoglobulin concentration may participate in the variability of NBCS [34].

A study by Fang and colleagues showed different outcomes of several NBCS sera tested on head and neck cell lines, fibroblasts, hybridomas, and epithelial cell lines. While a few NBCS sera were able to sustain cell proliferation at levels comparable to FBS, others supported only limited growth. In several cases, cells cultured with NBCS showed significant morphological changes, both between the different alternative sera and in comparison to FBS [26].

Nowadays, NBCS is used in cell culture and represents a lower-priced alternative for FBS [33]. Indeed, NBCS is widely used in the cryopreservation of spermatogonia and testis tissues [36].

#### Adult bovine serum

Adult bovine serum (ABS) is derived from adult animals (older than 1 year, but younger than 3 years) [37]. It is estimated that billions of tons of blood are wasted or not properly reutilized in the cattle industry [38]. For this reason, in many countries, attempts are made to recover this by-product to produce food additives, cosmetics, biological reagents, pet food, and also serum for cell culture [37]. Nonetheless, studies comparing the effects of ABS and FBS on different cell lines underlined some differences between the sera. First of all, macromolecules such as protein, albumin, glucose, and cholesterol levels are higher in ABS than in FBS (Table 1) [37]. Secondly, hormone levels are also higher in ABS [37], although these differences have not been conclusively linked to cell culture outcomes. A few studies report cell-line specific effects: male serum, rich in testosterone, enhances the proliferation of bovine and human myogenic satellite cells, promoting myoblast formation. On the other hand, the proliferation of breast cancer cells and immune cells is promoted by the presence of estrogen and estrone in female serum [39, 40]. These observations highlight the need to further investigate the role of hormonal differences in influencing cellular responses.

From a practical and economic standpoint, the use of ABS could partially reduce the cost of cell cultures because it is more readily available. In fact, while FBS production is limited to certain geographical areas, as previously discussed, ABS is obtained from byproducts of local slaughterhouses [41]. However, FBS is still preferable for most mammalian cell lines as it contains the components and factors critical for cellular growth, proliferation, differentiation, and low antibody content [42, 43].

**Table 1:** Estimed amounts of the different components of ABS and FBS. at Adapted from Yu et al. [37]

Component	ABS		FBS
	Female	Male	
Total protein	104 g/L	117 g/L	27 g/L
Albumin	51 g/L	56 g/L	18 g/L
Cholesterol	2.90 g/L	3.32 g/L	0.30 g/L
Glucose	2.66 g/L	1.92 g/L	0.87 g/L
Sodium	201 mmol/L	204 mmol/L	105 mmol/L
Potassium	8.0 mmol/L	9.9 mmol/L	8.2 mmol/L
Chloride	138 mmol/L	150 mmol/L	73 mmol/L
Calcium	10.4 mmol/L	9.4 mmol/L	10.0 mmol/L
Testosterone	1 ng/mL	6.2 ng/mL	0.4 ng/mL
Estradiol	105 pg/mL	23 pg/mL	18.6 pg/mL

#### Porcine and bovine platelet lysates

The role of platelets in stimulating cell growth and wound-healing processes has been studied since 1970. Ross and colleagues noted that platelets were necessary for stimulating smooth muscle cell proliferation [44], and ever since then, plasma has been obtained from various animal species, such as bovine or porcine [45]. This type of blood fraction is abundant in growth factors (PDGF and TGF-β) and adhesive factors [45]. Indeed, both the bovine and the porcine platelet lysates showed promising effects on cell growth and proliferation, as they were tested on several cell lines, including hybridomas and Vero cells (African green monkey kidney cell line) [45, 46].

Compared to FBS, platelet lysate can be considered a less expensive and more accessible alternative. However, platelet lysates are still affected by batch-to-batch variability and potential biological hazards, which vary according to the species of origin. Porcine platelet lysate also contains substantial levels of IgG, which should be minimized prior to cell culture applications. Additionally, incomplete separation during the initial centrifugation step may lead to erythrocyte contamination, and subsequent hemolysis can raise the hemoglobin content of the final lysate [45]. For these reasons, the use of porcine and bovine platelet lysates may have limited applicability as cell culture supplements, and they appear unlikely to fully replace FBS.

#### Bovine serum albumin

Throughout the years, researchers have attempted to substitute the whole serum with the most representative protein, the bovine serum albumin (BSA) [47]. This protein is produced by the liver and plays a central role in endogenous molecule transport, the maintenance of osmotic pressure, the reduction of reactive oxygen species (ROS) production, and the regulation of immune processes [48]. BSA can provide a suitable environment for cell growth as it binds growth factors, hormones, lipids, and amino acids and mediates their cellular internalization [17, 48]. It can also bind metal ions and scavenge free radicals (ROS) [17]. Rat, mouse, human cancer cell lines, and hybridoma cells can be cultured with a BSA-supplemented medium, as Yamane and colleagues reported in 1976 [49, 50]. However, the production cost of BSA remains a limiting factor, even when engineered microorganisms are employed, due to the large quantities needed [51]. Additionally, BSA can bind to potential toxins and other impurities, leading to contamination and batch-to-batch variability in the culture medium composition [3].

#### Bovine ocular fluid

Another kind of supplement obtainable from bovine specimens is bovine ocular fluid (BOF). It is collected from the eyes of calves less than 1.4 years old, within six hours from death at the latest. After sterilization using 70% ethanol, the eye’s fluid is filtered and centrifuged [52]. BOF contains several factors that might promote and sustain cell growth, such as vascular endothelial growth factor (VEGF), IGF, hypoxanthine, albumin, and fibronectin [53]. The lack of other important growth factors and macromolecules compels the use of BOF in combination with supplements, like sheep’s defibrinated plasma and human serum albumin. This serum replacement was demonstrated to be able to support the growth of WISH (human amniotic cell line), Vero, chicken embryonal fibroblasts, and human bone marrow fibroblasts [52]. However, the use of the BOF alone is still applicable for cryopreserrvation, especially the one derived from buffalos (BuOF) (Table 2). A study conducted in 2015 indicated that BuOF was proficient in cryopreserving CHO, HEK (human embryonic kidney cell line), C18-4 (mouse spermatogonia cell line), and mES cell lines (mouse embryonic stem cell line) [53]. BuOF is easily available, mainly in India, where it is a by-product of the slaughter of these animals. For this reason, its price is considerably lower than FBS (7-8-fold lower) [53, 54]. Furthermore, the absence of immunoglobulins in this immune-privileged organ might avoid some immunological-related effects in cell culture. However, being an animal derivative, BuOF is subject to variation in composition, depending on the age, sex, and origin of the animal [53].

**Table 2:** Estimated amounts of the different components of BuOF. Adapted from Varma et al. [53]

Component	Concentration (mg/L)
Total protein	5.0 ± 0.3
Albumin	2.0 ± 0.9
Cholesterol	225 ± 10
Glucose	565 ± 23
Vitamin C (Ascorbic acid)	72.7 ± 7.0

#### Horse serum

Horse serum (HS) is commonly used in cell culture as a feasible substitute for FBS, especially for equine cell lines. Using autologous or allogenic serum may prevent potential antigenicity associated with bovine proteins. On the other hand, there is a possible risk of transmission of species-specific viruses [55]. As for FBS, the batches of HS derive from numerous animals and are all tested for sterility and equine infectious anemia [56].

HS concentrations affect cell attachment, proliferation, and differentiation. In particular, high concentrations promote cell attachment but hinder cell proliferation and differentiation, which are allowed by low concentrations [57]. In addition, compared to FBS, it contains lower levels of growth factors and higher levels of immunoglobulins, limiting cell growth and proliferation [28]. Furthermore, being an animal-derived supplement itself, its use as a substitute for FBS may not align with the 3Rs principle.

#### Earthworm coelomic fluid

Recently, researchers have investigated the earthworm coelomic fluid as a feasible alternative to FBS in cell culture. The coelomic fluid (CF) is composed of an aqueous matrix, the plasma, and numerous cells involved in the innate immune system (the coelomocytes) [58]. This fluid is implicated in body movements, such as burrowing, respiration, innate immunity, and the transport of nutrients to every organ of *Perionyx excavates* [59]. The immune system of *P. excavates* relies principally on the innate system, hence the lack of antibodies in the coelomic fluid, which otherwise might interfere with cell culture [59]. Different methods are available to extract the CF: the worms generally respond to an extreme (cold or warm) temperature or electric discharges (about 5 Volts for 30 min) by extruding coelomic fluid. One of the benefits of CF is its easy availability: 1 kg of adult worm can allow the extraction of 140 mL of CF, and the process is repeatable within 10–15 days, allegedly without invasive harm to the animals. However, this could result in a difficult scalability of the production process [60].

Several studies have been conducted to define the bioactive molecules present in CF, among which there are proteins with hemolytic, cytotoxic, and antibacterial activity [58]. Indeed, the CF antiproliferative activity is currently being studied on cancer cell lines (breast, liver, brain, and oral cancer cell lines) [61]. In addition, CF lacks fibronectin, making cell attachment difficult. However, a few studies testing a heat-inactivated CF (HI-CF) on other cell lines, such as Vero, HeLa (human cervical carcinoma cell line), C2C12, and mouse primary fibroblasts, highlighted the possibility of using HI-CF as a substitute for FBS [59, 60]. To overcome the initial detachment of cells cultured with HI-CF, the supplement was enriched with growth and attachment factors and subsequently designated as formulated HI-CF (fHI-CF). All cells cultured with fHI-CF attached to the culture surface without early detachment, preserving their specific morphology. Growth rate of fHI-CF- and FBS-supplemented cells exhibited no differences, while that of cells cultured in a serum-free medium appeared reduced [60].

Concerning the biochemical profile of CF, Mathews and colleagues detected five vitamins present in CF, among which are riboflavin, cyanocobalamin, biotin, pantothenic acid, and nicotinic acid. All of these molecules play a crucial role in supporting cell growth and proliferation (Table 3) [60]. However, the comprehensive chemical profile of CF remains undefined, like that of FBS [59].

**Table 3:** Estimated amounts of several components and vitamins in heat-inactivated coelomic fluid. Adapted from Mathews et al. [60]

Component	Concentration
Sodium	113.1 mmol/L
Potassium	7.7 mmol/L
Chloride	262.3 mmol/L
Iron	1.02 ppm
Cholesterol	110 mg/L
Urea	37 mg/L
Creatinine	0.3 mg/L
Total protein	1.63 mg/L
Vitamin B_3_ (Nicotinamide)	93.87 µg/L
Vitamin B_7_ (Biotin)	6.895 µg/L
Vitamin B_12_ (Cyanocobalamin)	0.8295 µg/L

### Media obtained from humans

#### Human serum and human platelet lysate

During the last decades, the role of cell therapy and regenerative medicine has become increasingly central [62]. An immediate example is CAR-T cell therapy, based on the patient’s T-cell collection through blood samples and genetic engineering to target cancerous cells. This process involves the lymphocytes’ ex vivo culture and expansion, which can be influenced by the sera and serum substitute utilized [63]. As well as lymphocytes, mesenchymal stem cells and adipose tissue may require in vitro expansion and therefore can be influenced by the culture conditions [62]. In these cases, human serum (huS) and human platelet lysate (hPL) were successfully tested as alternatives to FBS. These human derivatives decrease the risks of zoonotic infections and immunological responses [64] since human sera of the AB blood group are used as they represent a universal donor [65]. Moreover, the collection process is regulated by blood bank procedures, making traceability and biological origin more attainable. The blood samples are tested for viral contamination and the biochemical profile. Generally, they are pooled together to compensate for batch-to-batch variability [64]. However, the use of huS and hPL in cell culture is still emerging, due to the need to meet international consensus on safety, quality, and production criteria [66].

#### Human serum albumin

Human serum albumin (HSA) is a globular protein and one of the most represented proteins in the plasma. Indeed, it has a plasma concentration of 35–50 g/L and an average life span of 19 days [67]. HSA is responsible for several important functions, both in the extra- and intracellular space [68]. For example, it is accountable for 80% of the osmotic pressure of blood, the maintenance of a suitable blood pH, and the binding of endogenous (copper, calcium) and exogenous (drugs) molecules [67]. Thanks to these intrinsic characteristics, not only is albumin currently studied as a drug carrier for multiple biomedical applications [69], but also as an alternative to FBS in cell culture medium [68]. Several studies reported the ability of HSA to sustain and improve the survival and growth of different cell lines, such as C2C12 [70], human embryonic stem cells, fibroblasts, and hybridoma cell lines [68]. However, due to the limited supply of HSA, recombinant DNA methods have been used to face the production request, frequently using yeast as a cell host. Nonetheless, the purification process and the ligands bound to purified HSA are the leading causes of batch-to-batch variability [68].

### Insect-based media

#### Silk protein sericin

Although silk production and trade have existed for centuries, its properties have only been extensively studied in recent decades. The most used type of silk in the trade market is the one produced by *Bombyx mori*, a species of insect belonging to the family of Bombycidae [71].

Usually, the sericin protein is discarded during the silk production process, resulting in a waste of about 50'000 tons of sericin worldwide [72]. As said above, the interest in sericin has grown in recent years, contributing to the recovery of this protein. This brings benefits from a social and environmental perspective to silk-producing countries, such as Brazil, India, and China [71]. In the biotechnology field, a silk-based bioink formulation was used as a 3D model to support platelet production from iPSC-derived and human primary megakaryocytes, showing promising results [73]. In addition, in recent years, silk sericin, or its hydrolysate, has shown encouraging results for cell culture and cryopreservation of different cell lines, such as HeLa cells or CHO cells [74, 75]. On the other hand, sericin shows mechanical fragility due to its structure and is significantly sensitive to pH changes and temperature fluctuations. The biological activity and functional properties of this protein strongly depend on its molecular weight and amino acid composition. Consequently, variability in extraction processes can result in molecular weight discrepancies, which may ultimately affect cell culture outcomes [76].

### Plant-based media

#### Algae extracts

The application of algae extracts in cell culture has gained considerable interest over the past decade [77]. Microalgae are organisms that synthesize lipids, carbohydrates, and amino acids very efficiently. These components are currently being evaluated as FBS alternatives. Okamoto and colleagues reported that glucose and amino acids extracted from microalgae can support mammalian cell cultivation [78]. Additionally, a study by Ng and coworkers investigated the bioactive components extracted from *Chlorella vulgaris*, termed chlorella growth factor (CFG), and demonstrated their capacity to promote the growth of mammalian cell lines under low serum conditions [79]. Finally, highly concentrated extracts of native proteins from *Galdieria sulphuraria* showed promising results in replacing FBS. A study conducted by Eisenberg and colleagues showed that heat-inactivated protein extracts were able to support CHO cell growth similarly to FBS. However, the production process shows batch-to-batch variability, suggesting a non-uniform protein composition in the extracts. It is also crucial to better characterize the biochemical profile of these algae extracts [77].

### Chemically defined media

To overcome FBS-associated batch-to-batch variability, chemically defined media for culturing eukaryotic cells have been studied since the 1950s. In those years, three key discoveries paved the way for the development of chemically defined media. McKeehan found that selenium is a fundamental trace nutrient, Hayashi and Sato successfully replaced serum with several combined hormones, and Guilbert and Iscove partially replaced serum with transferrin, selenite, albumin, and lecithin [80]. These findings led to the development of the first commercial medium supplement containing insulin, transferrin, and selenium (ITS), for serum-free cell cultivation in 1980. From this point on, several media have been developed and customized according to the needs of each cell line [80]. For example, Murakami and colleagues formulated a specific medium for hybridomas, containing ethanolamine [81]. Various chemically defined media were also developed for recombinant protein production, where the most commonly used cell line is CHO-K1. A few examples are Dulbecco’s Modified Eagle’s Medium with high glucose (DMEM), E-RDF [82], and Hybri-Care Medium (ATCC 46-X) [80].

Chemically defined media are also used in human iPSC cultivation. Chen and colleagues describe a simplified and more cost-efficient medium for human embryonic stem cells (ESCs) and iPSCs, called E8 medium. This defined medium reduces costs for cell culture, simplifies quality control, and should also facilitate the transfer from basic to clinical applications [83].

Another example of chemically defined serum is Ultroser G, commercially available for the culture of several cell lines [84]. It contains growth and adhesion factors, mineral elements, hormones, binding proteins, vitamins, and trypsin inhibitors, for a total protein concentration of 1.5 g/L [85]. A patent probably covers its semi-defined composition [29]. Ultroser G was evaluated for in vitro fertilization (IVF) on different cell lines, resulting successful on mouse embryos, but not on the human ones [86]. UltroserG was also used for the culture of various cell lines, among which were human skin fibroblasts, HeLa, Vero, rabbit chondrocytes, fetal rat hepatocytes, and Madin-Darby canine kidney cells (MDCK) [87].

Finally, Oredsson and colleagues developed a new defined medium, the Oredsson universal replacement medium. Its composition is entirely based on human proteins, either recombinant or derived from human tissues [88]. This medium is formulated on a DMEM/F12 base, supplemented with all the essential components following a published 14-step protocol. As it is not commercially available, it must be entirely prepared in-house. Consequently, initial costs can be substantial, as all components required need to be individually procured [89]. The Oredsson medium supports the long-term cultivation of different cell lines, both normal and cancerous, and their freezing and thawing. Moreover, it proved to be adequate in experimental settings, both in 2D and 3D [88].

Despite these promising results, the elevated cost of chemically defined media may still limit their broader adoption as a substitute for FBS in cell cultures.

## Milk as a substitute to FBS in cell culture

A major world challenge is food waste. The United Nations Food and Agriculture Organization (FAO) estimated that approximately 1.3 billion tons of food are wasted annually, representing one-third of all food supplies produced [90]. This wastage also entails a significant loss of energy, land, water, and labor employed for production [90]. Consequently, valorizing wasted food has become an urgent priority from both economic and environmental perspectives, and has attracted growing interest across several sectors, including biotechnology. In this context, protein hydrolysates and egg-derived extracts are being investigated as bioactive supplements to enhance the performance of serum-free culture media, showing promising results [91, 92].

Concerning dairy products, their production has witnessed a remarkable increase over the past decades [93]. In particular, milk yield increased from 53 Mt in 1994 to 887 Mt in 2021, with a 16.7-fold increase [94]. Notably, about 18.1% of the annual production of bovine milk is wasted due to insufficient storage, logistics, and expiration date issues [95]. Requalification of unused milk addressed to discarding responds to both unmet social and economic needs. Repositioning in the market of otherwise wasted milk through a circular economy process promotes environmental sustainability, together with the reduction of costs associated with disposal. Additionally, it does not require further animal exploitation, including fetal suffering and cow slaughter. In this way, otherwise wasted milk could be sold by stakeholders at a nominal price, as promoted in the UK by the ReFood project that offers a sustainable approach to recycling companies’ food waste by converting it into cleaner energy through a process of anaerobic digestion [96]. Thus, large retailers and small businesses alike could establish collaborations with biotech companies to valorize their food waste for biotechnological applications.

Government subsidies, as the Italian Law No. 166/2016 regulating the free or low-cost transfer of discarded food for social and anti-waste purposes, could encourage the reuse of otherwise wasted milk. Furthermore, when FBS is sourced from approved bovine facilities [8], a similar controlled supply chain could be established within the dairy sector, enabling farms to collect, preserve, and transfer surplus milk to biotech companies. This approach would reflect the existing FBS production model, where raw material is processed and quality-audited before commercialization [97].

### Milk and its composition

Milk is the fluid produced by the mammary glands of female mammals to nourish their offspring [98]. It is known to be an essential part of life and development thanks to its nutritional components and the richness of nutrients, amino acids, and nitrogen sources [99, 100]. For these characteristics, its use as a cell culture supplement is a possible way to reduce or even replace FBS [101], while also reducing food waste. Milk includes up to 2000 components [102], such as lipids, vitamins, minerals, lactose, hormones, immunoglobulins, and proteins [103]. Not only do they meet nutritional demands, but they also exert antioxidant, anti-inflammatory, and immunostimulatory activities [104]. Some of these milk components are produced by the rough endoplasmic reticulum of the mammary gland (lactose, lipids, proteins), while others permeate from blood (vitamins, water, immunoglobulins, and some hormones) [98].

**Fig. 3:**
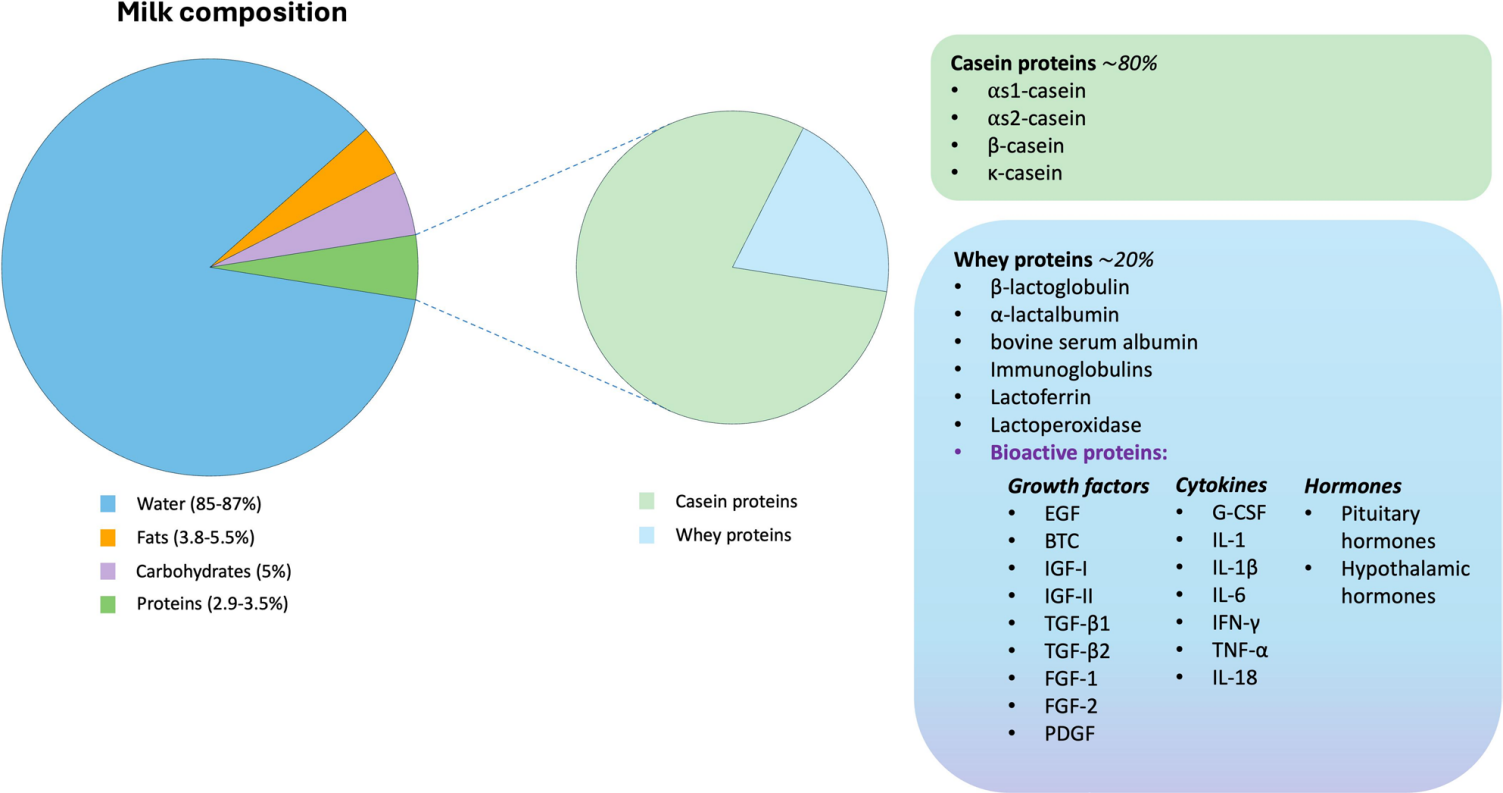
Milk composition and respective percentages. Milk is composed of water (85–87%), fats (3.8-5.5%), carbohydrates (5%), and proteins (2.9–3.5%). The latter can be divided into casein proteins (insoluble, 80%) and whey proteins (soluble, 20%). Whey proteins are a heterogeneous group, comprising bioactive molecules such as growth factors, cytokines, and hormones that could play a key role in cell growth and proliferation

The macronutrients in milk are water (85–87%), fats (3.8–5.5%), carbohydrates (5%), and proteins (2.9–3.5%) [105] (Fig. 3).

### Milk fats

Milk fat globules (MFG) represent the most important source of fats and many bioactive components in milk. Fats constitute a great source of energy (about 9 kcal/gram) and are a viable carrier of fat-soluble vitamins (A, D, E, and K). MFG is defined by a membrane composed of polar lipids and proteins (the milk fat globule membrane, MFGM), and a core containing triglycerides and cholesterol esters. MFGM is essential to avoid the degradation of triglycerides by the enzyme lipase [98]. Milk polar lipids provide choline, ethanolamine, and polyunsaturated fatty acids, crucial for the growth and plasticity of tissues [106]. For example, choline is a precursor for the biosynthesis of many membrane components, and sphingomyelin and its metabolites show regulating effects on cell proliferation and cell survival [107].

### Milk carbohydrates

Lactose is the main carbohydrate in milk and is a disaccharide composed of D-glucose (α- or β-D-glucose isoforms) and β-D-galactose, linked by a β-1,4-glycosylic bond [108]. The equilibrium between these two isoforms depends on several parameters, such as temperature, pH, and lactose concentration. Lactose is an important energy source, responsible for the osmotic equilibrium between the alveolar lumen of the mammary gland and blood. Lactic acid bacteria can ferment lactose into lactic acid, which is accountable for the pH decrease of milk. The correct management of this process is crucial for producing yogurt and cheese [98].

### Milk proteins

The bovine milk proteins can be divided into two major groups: caseins (representing 80% of the total protein content, insoluble) and whey proteins (20%, soluble), both showing antioxidant activities [104, 109]. These proteins are activated by enzymatic hydrolysis [100]. The first group includes αs1-, αs2-, β-, and k-caseins. Their amino acidic sequence and structure are responsible for the antioxidant activity, especially tryptophan, tyrosine, histidine, and proline residues [104]. The casein micelle is generally composed of proteins for 94% and minerals for 6% [98]. The protein hydrolysis changes the structure and increases the ROS scavenger activity and protection against lipid peroxidation, as demonstrated in the Caco-2 cell line (human adenocarcinoma colon cancer cell line) [104].

The casein precipitation, induced by rennet or acid coagulation, leads to the formation of milk whey. Milk whey comprises around 85–95% of total milk volume and 55% of total milk components. It is rich in proteins, especially those still soluble after casein precipitation (Table 4) [110]. A globular structure characterizes whey proteins, which include β-lactoglobulin (β-LG), α-lactalbumin (α-LA), bovine serum albumin (BSA), immunoglobulins, lactoferrin, and lactoperoxidase [101]. As well as casein proteins, whey proteins exert antioxidant activity, chelate metals, and scavenge free radicals [104]. In addition, milk whey contains hormones, growth factors, and other molecules that can support cell growth and proliferation. A study published in 2019 compared the biochemical profiles of FBS and bovine whey proteins (BWP), which were similar. However, the ultrafiltration of BWP resulted in better cell growth of at least two different cell lines, CHO-K1 (Chinese hamster ovary cancer cell line) and Jurkat E6.1 (a human lymphoblastic leukemia cell line). This process allows the concentration of BWP proteins, enhancing their cell growth-supporting properties [101].

Bovine milk whey is more suitable for culturing cell lines than casein proteins. Few references in the literature report the use of casein proteins as a supplement for culture media, for example for insect cell lines [111]. They find greater use in non-biotechnology fields: the stickiness and viscosity of casein proteins make them suitable for manufacturing glues, films, and biomaterials [112].

On the other hand, bovine milk whey contains bioactive proteins that can support cell growth and proliferation. The β-LG protein is produced by the mammary gland and represents 58% of whey proteins [113]. Its structure suggests the capacity to bind small hydrophobic ligands, such as vitamin D, vitamin A, palmitic acid, and fatty acids [102, 114]. This whey protein accounts for several biological activities, including the prevention of pathogen adhesion and anticarcinogenic effects [115]. Moreover, it represents a great source of peptides with different biological activities and essential amino acids, among which are branched-chain amino acids (BCAA) [113]. Liu and colleagues demonstrated that β-LG is accountable for 50% of milk antioxidant activity, but this characteristic is strongly reduced in heated raw milk due to a conformational change in the protein structure [116]. Moreover, Tai and his group showed that β-LG is the main protein of whey milk that promotes cell growth and proliferation through the binding of the β-LG receptor, which is a membrane IgM [114].

The second most important whey protein is α-LA, representing about 20% of total whey proteins [115]. It does not contain a free -SH group, therefore it is rather heat stable [113]. In addition, α-LA has a key role in the biosynthesis of lactose, as it catalyzes the final step of the process [113]. Moreover, α-LA is rich in BCAA and in tryptophan and cysteine, which are the precursors of serotonin and glutathione, respectively [115].

Bovine serum albumin (BSA), differently from α-LA and β-LG, is not synthesized by the mammary gland, but it permeates from the blood into the milk. It represents about 6% of the total whey proteins and has specific binding sites for hydrophobic molecules. In blood serum, it is a carrier for fatty acids and it might preserve this function in milk as well [98, 113]. Its efficacy has already been demonstrated with different mammalian cell lines, as reported by Yamane and colleagues [48, 49].

Immunoglobulins (Igs) represent the largest and most heterogeneous group of whey proteins [98]. They are present in the milk of all lactating species. Igs can be found at the highest concentration in the colostrum (40–200 g/L), as they provide primary passive immune protection to the newborn calf until the infant can produce their own [113, 117]. In particular, in bovine colostrum, Igs account for up to 80% of the whole protein content [115]. However, they represent only 2% of total proteins and 10% of total whey proteins in milk. Three classes of Igs are detectable in milk: IgA, IgG, and IgM. Except for the latter, they are all selectively transported from the blood to the mammary gland, and this explains the highest concentration of immunoglobulins in colostrum [113]. Immunoglobulins are thermolabile (for temperatures > 75 °C) and exposure to heat can induce conformational changes in their structures. A typical pasteurization process results in the preservation of 25–75% of the total Ig concentration compared to raw milk, while ultra-high temperature treatment (UHT) processes result in little to no detectable Ig levels [118]. Lower levels of Igs in cell culture media are preferable to avoid immunological responses and possible interference with cell growth.

Lactoferrin (LF) is an iron-binding glycoprotein present in colostrum and milk at concentrations of 1.5 g/L and 0.02–0.35 g/L, respectively. In addition to iron, it can also bind copper and other metal ions present in cell culture media, potentially affecting cellular growth [113, 119]. The binding of iron molecules results in antimicrobial properties, as it impoverishes bacteria of an essential element for their growth [120]. LF has several properties, including antimicrobial, antioxidative, anti-inflammatory, anti-cancer, and immune regulatory properties [115].

Lastly, lactoperoxidase (LP) is an enzyme present in colostrum and milk at concentrations of 11 to 45 mg/L and 13 to 30 mg/L, respectively. It constitutes about 0.5% of total whey proteins [113]. LP catalyzes a reaction that involves hydrogen peroxide and thiocyanate ions and produces hypothiocyanite ions, responsible for the antimicrobial actions [113, 115]. The lactoperoxidase system plays a key role in the innate immune system by killing bacteria in milk [113].

**Table 4:** Estimated amounts of the different components in whole milk, milk whey, and FBS. * The conversion was calculated considering the molecular weight of the element

Component	Whole milk		Milk whey (Acid)		FBS	
	Concentration	Reference	Concentration	Reference	Concentration	Reference
Lactose	4.80–5%	[121]	44–46 g/L	[110]	–	
Total protein	3.1–3.48%	[121]	6.0–8.0 g/L	[110]	27 g/L	[37]
Calcium	0.971–1.42 g/L	[121]	1.2–1.6 g/L	[110]	10 mmol/L (0.401 g/L)*	[37]
Phosphate	2.30 g/L	[98]	2.0–4.5 g/L	[110]	–	
Magnesium	0.086–0.118 g/L	[121]	48 mg/kg	[122]	–	
Potassium	1.509–1.896 g/L	[121]	962 mg/kg	[122]	8.2 mmol/L (0.321 g/L)*	[37]
Sodium	0.366–0.476 g/L	[121]	225 mg/kg	[122]	105 mmol/L (2.414 g/L)*	[37]
Chloride	1.10 g/L	[98]	1.1 g/L	[110]	73 mmol/L (2.59 g/L)*	[37]
Bovine serum albumin	0.41–0.49 g/L	[121]	0.4 g/L	[109]	18 g/L	[37]
Iron	0.26–0.67 mg/L	[121]	–	–	–	
Testosterone	0.10 ± 0.01 ug/kg	[123]	–	–	0.4 ng/mL	[37]
17β-Estradiol	26.75–52.91 pg/mL	[124]	–	–	18.6 pg/mL	[37]
Progesterone	2.14–3.23 ng/mL	[124]	0.209–1.01 ng/mL	[125]	–	
Lactoferrin	0.02–0.35 g/L	[113]	0.1 g/L	[109]	–	
Lactoperoxidase	13–30 mg/L	[113]	30 mg/L	[109]	–	
α-Lactoalbumin	1.0–1.5 g/L	[126]	1.2 g/L	[109]	–	
β-Lactoglobulin	3.2–4.10 g/L	[127,128]	1.3 g/L	[109]	–	

### Milk bioactive molecules

In addition to the macromolecules described above, milk and milk whey contain many bioactive components that play a key role in mitogenic processes and mediator activities. These molecules can exert their biological activities even at very low concentrations (< 0.001 g/L) compared to other components previously cited, such as lactoferrin (0.02–2 g/L) and immunoglobulins (0.8 g/L) [129]. It is now recognized that all these elements can modulate physiological functions and could positively impact human health. For this reason, in the past decade, researchers have focused on identifying these components and their properties [129]. Growth factors, hormones, and cytokines can be found in bovine milk and colostrum [130] and can influence growth, differentiation, and repair processes in different types of cells [131]. Among the growth factors, EGF, betacellulin (BTC), IGF-I, IGF-II, TGF-β1, TGF-β2, fibroblast growth factors (FGF-1 and FGF-2), and PDGF were identified and quantified in bovine colostrum and milk (Table 5). Their concentration reaches the highest value in the first hours of calving, hence in colostrum, and generally decreases in the following days [131]. Briefly, IGF-I and IGF-II are important regulators of cellular differentiation, cellular growth, protein and carbohydrate metabolism. They circulate bound to proteins of a family of high-affinity insulin-like growth factor binding proteins (IGFBPs). These proteins control the efflux of IGFs, prolong their half-lives, regulate their metabolic clearance, and modulate the interaction between IGFs and their receptors [132]. Among these binding proteins, IGFBP-2 and IGFBP-3 are the most present in bovine colostrum, and their concentrations decrease 3 days after calving [131]. TGF-βs play a key role in embryogenesis, tissue repair, inflammatory processes, and the control of the immune system [130]. This family includes various factors characterized by specific activities that vary depending on the cell type [131]. The main form in bovine milk is TGF-β2 (85%) [129].

**Table 5:** Estimated amounts of the growth factors in bovine colostrum and bovine milk

Growth factor	Concentration in bovine colostrum	Concentration in bovine milk	Principal source	Primary activity	References
EGF	4–324.2 ng/mL	< 2 ng/mL < 2 µg/mL	Different tissues and body fluids	Stimulation of the proliferation of epidermal, epithelial and embryonic cells	[131] [133] [134]
BTC	2.3 ng/mL	1.9 ng/mL	Different tissues and body fluids	Promotion of wound healing	[131]
IGF-I	49–2000 ng/mL 50–2000 µg/mL	2–150 ng/mL < 10 µg/mL 10 µg/mL	Liver	Stimulation of the proliferation of many types of cells	[131] [133] [134]
IGF-II	150–600 ng/mL 200–600 µg/mL	2–107 ng/mL < 10 µg/mL	Different cells	Stimulates the differentiation of several kinds of cells	[131] [133]
TGF-β1	12.4–42.6 ng/mL	0.8–3.7 ng/mL 4.3 µg/mL	Different cells	Regulation of cell proliferation, tissue repair and immune responses	[131] [133]
TGF-β2	150–1150 ng/mL	13–66 ng/mL	Platelets and other cells	Stimulation of the cell growth, embryogenesis, wound healing, immune system response	[131] [133]
FGF1	–	< 4 ng/mL			[131]
FGF2	–	< 4 ng/mL	Different kinds of cells	Stimulation of wound healing, angiogenesis, proliferation, differentiation and survival of various kinds of cells	[131]

For what concerns cytokines, GM-CSF, interleukin 1 (IL-1), interleukin 1β (IL-1β), interleukin 6 (IL-6), interferon g (IFN-g), tumor necrosis factor α (TNF-α), and interleukin 18 (IL-18) were detected in bovine colostrum and milk [131].

The hormones found in milk generally derive from maternal circulation and are secreted in milk through active transport within the mammary gland [135]. Their structure and activities can remain unchanged or can be modified through glycosylation, phosphorylation, or proteolysis [136]. In general, hormones have a key role in stimulating and maintaining cell functions. For example, insulin promotes the uptake of glucose and other nutrients and the maintenance of differentiation [137].

The main molecules are gonadal, pituitary, adrenal, and hypothalamic hormones (Table 6). Among gonadal hormones, we can find progesterone and estrone. Progesterone is absent in colostrum, and its concentration levels increase 15 days after parturition. Estrone is the primary estrogen hormone found in milk. In whey, it is bound to proteins up to 48% of its total [135]. Glucocorticoids and corticosterone are the principal adrenal gland hormones found in milk. The corticosterone concentration generally increases during lactation, while the glucocorticoid concentration decreases gradually and significantly during the same period. Glucocorticoids may regulate the production of lactose by reducing the glucose uptake, which is an important substrate for lactose production [135]. Prolactin is a pituitary hormone found in higher concentrations in colostrum than in milk. Part of its content is found associated with milk fat globules, and one of its activities could be stimulatory regarding lactation [135].

**Table 6:** Estimated amounts of the hormones in whole milk. Adapted from Jouan et al. [135]

Hormones	Ranges reported in milk
**Gonadal Hormones**	
Progesterone	2–20 ng/mL
Estrogens	5–10 pg/mL
Androgens	0–50 pg/mL
**Adrenal gland hormones**	
Glucocorticoids	0–50 ng/mL
**Pituitary hormones**	
Prolactin	5–200 ng/mL
Growth hormone	0–1 ng/mL
**Hypothalamic hormones**	
Gonadotropin-releasing hormone	0.5–3.0 ng/mL
Luteinizing hormone-releasing hormone	0.5–3.0 ng/mL
Thyrotropin-releasing hormone	0–0.5 ng/mL
Somatostatin	10–30 ng/mL

Lastly, milk also contains a variety of vitamins (Table 7) that serve a critical function in supporting proper cellular growth. It is reasonable to assume that pantothenate, riboflavin, folic acid, thiamine, and nicotinamide are essential for the optimal culture of most cell lines [137, 138]. They can be classified as lipophilic vitamins (A, D, E, and K) if they contain hydrophobic groups, and can be found in whole milk, cream, butter, and cheeses. On the other side, hydrophilic vitamins (B_1_, B_2_, B_3_, B_5_, B_6_, B_8_, B_9_, B_12_, and C) are soluble in water and can be found in skim milk. Several factors can contribute to their variability in concentration, such as diet, products, and sensitivity to temperature, light, and O_2_ [139].

**Table 7:** Estimated amounts of the vitamin concentrations in whole milk. Adapted from *Handbook of Food Chemistry* [98]

Vitamin	Concentration (mg/L)
A (Retinol)	0.4
D (Calciferol)	0.001
E (Tocopherol)	1.0
B_1_ (Thiamine)	0.4
B_2_ (Riboflavin)	1.7
B_3_ (Nicotinamide)	1
B_5_ (Pantothenic acid)	3.5
B_6_ (Pyridoxine)	0.6
B_7_ (Biotin)	0.03
B_9_ (Folic acid)	0.05
B_12_ (Cyanocobalamin)	0.005
C (Ascorbic acid)	20

## Milk in cell culture

For over three decades, studies have been conducted to test the potential of whey or milk derivatives in cell cultures, ranging from partial to complete replacement of FBS [140].

Capiaumont and colleagues tested a medium containing 9% BWP and 1% FBS on CHO-K1 cells to reduce FBS usage. The whey was prefiltered through a 0.1 μm membrane, and the retentate was subsequently heated at 56 °C for 1 h. A second filtration through a 30 kDa membrane was used to concentrate the whey fraction, which was finally sterilized using a 0.22 μm membrane. After adaptation, the cell growth was comparable to that obtained with 5% FBS and even superior to that observed with 1% FBS [141]. In addition, a study conducted by Boga and co-workers investigated the possibility of using milk or whey proteins to minimize the use of FBS in human placenta mesenchymal stem cell (hPMSC) culturing. The results were encouraging, as the milk- and whey-supplemented media preserved the stemness of hPMSCs in vitro. However, the growth of these cells cultured with milk or whey alone was not sufficient. Culture medium supplemented with 1% milk in addition to FBS showed a proliferation almost equal to the control, highlighting the possibility of reducing the use of serum in culture media [142].

Derouiche and colleagues evaluated the growth of hybridoma cell lines A_49_ and F_34_ cultivated with media containing whey fractions or FBS. Whey fractions supported cell growth, and antibody production (assessed on the 7th day for A_49_ or the 9th day for F_34_) was comparable to that obtained with FBS-supplemented cultures. However, a minimum of 1% FBS was required for long-term cell culturing, achieving similar performances to cultures grown with 10% FBS [143].

Similar results also emerged from a work by Paradkar and colleagues. BWP with a minimal amount of FBS effectively supported cell growth of CHO-K1 and Jurkat E6.1 cell lines. However, enrichment of BWP with hPL successfully sustained the cell growth in vitro, leading to a complete replacement of FBS in cell culture [101].

Finally, several studies report a successful complete substitution of FBS with milk whey or other derivatives [140, 144–147].

In a work from Pakkanen, a mixture composed of an ultrafiltrate fraction of bovine colostrum (6.7%), adult bovine serum (ABS, 1%), and human holo-transferrin (5 mg/L) was tested for the cultivation of CHO-K1 and Vero cell lines. This medium permitted the long-term cultivation of these cell lines and could also be used as a freezing medium. In addition, it has proven to be more economically advantageous than FBS [145].

Belford and colleagues have further evaluated studies on epithelial and fibroblast cell lines of different species, using a mitogenic fraction derived from bovine milk. The results highlighted that, with the addition of attachment-promoting factors, the milk fraction was able to provide the necessary growth factors to sustain cell growth [146].

A work by Purup and colleagues tested different milk fractions, derived from distinct stages of lactation, on the FHs 74 cell line (human intestinal cell line). In addition to all being effective in promoting cell growth, the growth-promoting activity gradually increased as the stage of lactation advanced. This increase is probably related to changes in the concentration of growth factors, such as IGF-I, TGF-β1, and TGF-β2 [147].

The replacement of FBS in cell culture is a key aspect of in vitro meat cultivation, as the potential presence of pathogenic factors poses safety issues, especially for products destined for human consumption. Consequently, in recent years, increasing attention has been devoted to the transition toward serum-free cell culture systems in this area of research as well [140]. Sundaram and colleagues tested whey proteins on the C2C12 cell line, a model commonly used in vitro meat cultivation. The whey proteins effectively promoted cell proliferation and maintained cell membrane integrity, suggesting their potential as an alternative for FBS [140]. The final goal for cultivated meat should be complete independence from animal-derived products, with milk-derived supplements potentially providing a transitional solution toward fully animal-free cell culture [11].

Additionally, some milk derivatives can be used in the formation of scaffolds required to support cell growth for cultivated meat. Tahir and colleagues highlighted the advantages of incorporating whey protein isolate and β-lactoglobulin into hydrogel networks, supporting cell adhesion and proliferation [144].

The studies cited above report promising results. Nonetheless, milk-derived supplements used in culture media remain subject to batch-to-batch variability, and their animal origin constitutes a potential limitation. At the same time, their application in cell culture presents important economic, environmental, and ethical considerations, as they may contribute to the circular economy and offer a more cost-effective alternative compared to conventional supplements.

## Conclusion

Cell cultures play a pivotal role in scientific research, drug and vaccine production, and cell factory activities. Over the past few decades, their widespread use has caused the consumption of more than half a million liters per year of FBS, raising significant ethical, economic, and sustainability issues. Finding a substitute for FBS in culture media is urgent to guarantee the sustainability of the cell culture-based approaches.

In this review, we examined various alternatives to FBS, highlighting their advantages and limitations. Among these, milk-derived additives appear particularly promising due to their compositional similarity to FBS, lower production costs, and ability to address ethical issues associated with animal-derived products, with studies reporting data ranging from significant reductions to full replacement of FBS. The use of milk derivatives does not overcome some of the limits associated with FBS, such as the animal origin, the batch variability, and the presence of contaminants. Further efforts will be required to advance the transition toward animal-free media for cell cultures. However, milk-derived supplements could serve as an intermediate step to solve some of the issues related to FBS use. Collaborations between biotech companies and retailers may represent a way to repurpose surplus milk and limit food waste. Nonetheless, milk is subjected to strict controls as marketed primarily for food use, and, unlike FBS, can be sourced within Europe, ensuring compliance with European Community standards across all batches and reducing the environmental impact associated with long-distance transportation. Finally, it would reduce the waste of milk withdrawn from the shelves close to expiry and lead to the development of a new industrial sector. Further research and standardization are needed to provide more sustainable and ethically responsible cell culture systems.

## Data Availability

Not applicable.

## Abbreviations

3Rs: Replacement, Reduction, and Refinement
ABS: Adult bovine serum
BCAA: Branched-chain amino acids
BOF: Bovine ocular fluid
BSA: Bovine serum albumin
BTC: Betacellulin
BuOF: Buffalo ocular fluid
BVDV: Bovine viral diarrhea virus
BWP: Bovine whey proteins
CF: Coelomic fluid
DMEM: Dulbecco’s Modified Eagle’s Medium
EGF: Epidermal growth factor
EIA: Equine infectious anemia
ESCs: Human embryonic stem cells
EVs: Extracellular vesicles
FAO: Food and Agriculture Organization of the United Nations
FBS: Fetal bovine serum
FGF-1/FGF-2: Fibroblast growth factor 1 and 2
fHI-CF: Formulated heat-inactivated coelomic fluid
GM-CSF: Granulocyte colony-stimulating factor
HI-CF: Heat-inactivated Coelomic fluid
HSA: Human serum albumin
hPL: Human platelet lysate
hPMSC: Human placenta mesenchymal stem cell
HS: Horse serum
huS: Human serum
IFN-γ: Interferon γ
IGFBPs: Insulin-like growth factor binding proteins
IGF-I/IGF-II: Insulin like growth factor I and II
Igs: Immunoglobulins
IL-1/IL-1β: Interleukin 1 and interleukin 1β
IL-18: Interleukin 18
IL-6: Interleukin 6
iPSC: Induced pluripotent stem cells
IVF: In vitro fertilization
LF: Lactoferrin
LP: Lactoperoxidase
mES: Mouse embryonic stem cells
MFG/MFGM: Milk fat globule/milk fat globule membrane
NBCS: Newborn calf serum
PDGF: Platelet-derived growth factor
ROS: Reactive oxygen species
TGF-β1/TGF-β2: Transforming growth factor beta 1 and 2
TNF-α: Tumor necrosis factor α
UHT: Ultra-high temperature
VEGF: Vascular endothelial growth factor
α-LA: α-lactalbumin
β-LG: β-lactoglobulin
